# Higher prevalence of harbouring *BCR::ABL1* in first-degree relatives of chronic myeloid leukaemia (CML) patients compared to normal population

**DOI:** 10.1186/s12885-024-12102-2

**Published:** 2024-06-14

**Authors:** Jew Win Kuan, Anselm Ting Su, Sai-Peng Sim, Siow Phing Tay

**Affiliations:** 1 Department of Medicine, Faculty of Medicine and Health Sciences (FMHS), Universiti Malaysia Sarawak (UNIMAS), Jalan Datuk Mohammad Musa, Kota Samarahan, Sarawak, 94300 Malaysia; 2 Department of Community Medicine and Public Health, FMHS, UNIMAS, Sarawak, Kota Samarahan Malaysia; 3 Department of Para-Clinical Sciences, FMHS, UNIMAS, Sarawak, Kota Samarahan Malaysia; 4 Department of Pathology, FMHS, UNIMAS, Sarawak, Kota Samarahan Malaysia

**Keywords:** Chronic myeloid leukaemia, *BCR:ABL1*, Pre-clinical, Asymptomatic, Normal, Familial

## Abstract

**Background:**

The role of familial influence in chronic myeloid leukaemia (CML) occurrence is less defined. Previously, we conducted a study to determine the prevalence of harbouring *BCR::ABL1* in our local adult normal population (designated as Study^N^). We present our current study, which investigated the prevalence of harbouring *BCR::ABL1* in the normal first-degree relatives of local CML patients (designated as Study^R^). We compared and discussed the prevalence of Study^R^ and Study^N^ to assess the familial influence in CML occurrence.

**Methods:**

Study^R^ was a cross-sectional study using convenience sampling, recruiting first-degree relatives of local CML patients aged ≥ 18 years old without a history of haematological tumour. Real-time quantitative polymerase chain reaction standardised at the International Scale (*BCR::ABL1*-qPCR^IS^) was performed according to standard laboratory practice and the manufacturer’s protocol.

**Results:**

A total of 96 first-degree relatives from 41 families, with a mean age of 39 and a male-to-female ratio of 0.88, were enrolled and analysed. The median number of relatives per family was 2 (range 1 to 5). Among them, 18 (19%) were parents, 39 (41%) were siblings, and 39 (41%) were offspring of the CML patients. Study^R^ revealed that the prevalence of harbouring *BCR::ABL1* in the first-degree relatives was 4% (4/96), which was higher than the prevalence in the local normal population from Study^N^, 0.5% (1/190). All four positive relatives were Chinese, with three of them being female (*p* > 0.05). Their mean age was 39, compared to 45 in Study^N^. The *BCR::ABL1*–qPCR^IS^ levels ranged between 0.0017%^IS^ and 0.0071%^IS^, similar to Study^N^ (0.0023%^IS^ to 0.0032%^IS^) and another study (0.006%^IS^ to 0.016%^IS^).

**Conclusion:**

Our study showed that the prevalence of harbouring *BCR::ABL1* in the first-degree relatives of known CML patients was higher than the prevalence observed in the normal population. This suggests that familial influence in CML occurrence might exist but could be surpassed by other more dominant influences, such as genetic dilutional effects and protective genetic factors. The gender and ethnic association were inconsistent with CML epidemiology, suggestive of a higher familial influence in female and Chinese. Further investigation into this topic is warranted, ideally through larger studies with longer follow-up periods.

**Supplementary Information:**

The online version contains supplementary material available at 10.1186/s12885-024-12102-2.

## Background

Chronic myeloid leukaemia (CML) is a myeloproliferative neoplasm that originates from haematopoietic stem cells and is characterised by a chromosomal translocation t(9;22)(q34;q11.2), which forms the Philadelphia chromosome (Ph) containing the *BCR::ABL1* fusion gene [1]. Evidence strongly indicates that BCR-ABL1 tyrosine kinase, the translated protein of *BCR::ABL1*, is the primary driver of CML. Diagnosis of CML requires detection of the Ph and/or *BCR::ABL1*, along with the clinical features, leucocytosis, and other laboratory findings. Untreated CML typically progresses through three phases: chronic phase (CP), marked by leucocytosis (total white blood cell count (TWC) ≥ 12 × 10^9^/L); acceleration phase (AP); and blast crisis (BC) [1]. The introduction of tyrosine kinase inhibitor (TKI) targeting BCR-ABL1 has significantly improved prognosis. However, approximately 7% [2] to 18% [3] of CP patients progress to AP/BC despite treatment with imatinib, the first-generation TKI. In the latest World Health Organisation Classification (5^th^ edition), AP is omitted in favour of an emphasis on high-risk features associated with CP progression and resistance to TKI [4]. 

For identifying CML-related laboratory features at low cancer load, the most sensitive test is polymerase chain reaction (PCR) detecting *BCR::ABL1*, followed by cytogenetic test detecting Ph, and then, full blood count (FBC) primarily identifying leucocytosis. Clinical features of CP e.g., constitutional symptoms and splenomegaly, typically appear thereafter. *BCR::ABL1* transcript can be classified into major (M-BCR), minor (m-BCR), and micro (µ-BCR) depending on the breakpoint in *BCR*. M-BCR is the predominant transcript type in CML and the only one that has been standardised at International Scale (IS). IS-standardised real-time quantitative polymerase chain reaction (qPCR) for *BCR::ABL1* (*BCR::ABL1*-qPCR^IS^) allows direct comparison of the *BCR::ABL1* M-BCR level across different laboratories. When a CML patient achieves complete cytogenetic response (no detectable Ph) after starting TKI, the approximate equivalent *BCR::ABL1* is around 1%^IS^ [5]. 

There have been cases, of which Ph was detected incidentally in the absence of clinical features or leucocytosis, prompting a need of a new entity, designated as pre-clinical CML (pre-CP) [6] in this manuscript. In order to exclude those pre-CP cases without leucocytosis due to concurrent disease or medication usage that causes cytopaenia, we continue to use a criterion used by Kuan et al. [6] i.e., Ph was positive in < 75% metaphases. Pre-CP, at the median Ph of 35% (range 10-75%), often progresses to CP within a mean duration of 16 months (range 3 to 48) (Supplementary Table 1) [7–17]. The duration is supported by Hauser RG et al. [18] who investigated 1,623 patients underwent M-BCR testing for CML diagnosis and had at least six consecutive years of FBC with differentials prior to CML diagnosis. Among them, 6.2% were found to be M-BCR positive. This study revealed that the minimum basophil percentage over the previous year and the minimum TWC over the previous three years could predict a positive *BCR::ABL1* M-BCR test result. Before the CML CP diagnosis, abnormalities in TWC and basophil percentage could persist for a year or more.

Simultaneously, some normal subjects were found harbouring *BCR::ABL1* [19]. Most studies on normal subjects harbouring *BCR::ABL1* used convenience sampling. Thus, the results could not be inferred to a normal population. About 8% of the normal subjects/population harboured *BCR::ABL1* M-BCR (Supplementary Table 2) [20–31]. While pre-CP often progresses to CP, there was no reported progression to pre-CP or CP from normal subjects/population harbouring *BCR::ABL1*, probably because most studies did not report follow-up information on the positive subjects [19]. However, a lower *BCR::ABL1* load might be a reason. Most studies did not use *BCR::ABL1*-qPCR^IS^ making comparison across studies and further deduction difficult. Pre-CP is a subset of the normal subjects/population harbouring *BCR::ABL1*. We hypothesized that a considerable yet undetermined genetic abnormality load, maybe around 0.1%^IS^, is needed for CML occurrence.

Subsequently, it is interesting to investigate healthy relatives of CML patients who are a subset of normal population. CML is generally considered non-inheritable, but reported familial CML cases suggest genetic susceptibility associated with heritage (Supplementary Table 3) [32–45]. Familial CML is rare, partly attributed to the low incidence rate of CML. In 2017, global age-standardized incidence rate (ASIR) of CML typically ranged between 0.26 and 0.75 per 100,000 population [46]. Our local data also reported a low ASIR of 0.5 per 100,000 population from 1996 to 2015 [47]. However, the prevalence of CML is increasing due to the drastic improvement in survival with TKI. The aetiology of *BCR::ABL1* and non-*BCR::ABL1*-related disease driver remain poorly understood. We hypothesize the occurrence of CML may be sporadic, related to genetic susceptibility, or combination of both, intertwining with environmental elements and protective genetic factors. Notable risk factor is ionized radiation [48]. Other suggested risk factors are benzene [49], obesity [50], and smoking [51]. Familial CML cases highlight genetic susceptibility in some CML patients that are worth further studying. Human leucocyte antigen [52–57] and single nucleotide polymorphisms [58] were suggested but a conclusive answer is not yet apparent. The reported familial CML cases were more common in first-degree relatives, noting that cases in more distance relative cases are likely more under-reported than first-degree relatives.

Only a few non-case report studies [33, 59–62] have examined risk of getting CML in relatives of CML patients. An earlier study by Gunz FW et al. [33] in Sydney, Australia, investigated 909 leukaemia patients, including 119 with CML, using interviews and tracing techniques. The study showed that the incidence of leukaemia was 2.8-3.0 times higher among first-degree relatives and about 2.3 times higher among more distant relatives compared to the expected rates. However, the study noted an unusually low proportion of CML cases among first-degree relatives, with only one instance of concordance (CML-CML). Another study by Hasle H and Olsen JH [62] investigated cancer in relatives of children with myelodysplastic syndrome, acute myeloid leukaemia and CML in Denmark. There were 37 first-degree relatives, as well as 7 second- and third-degree relatives of eight CML children in the study. They found no case of CML or increase risk of neoplasms in the relatives. Two consecutive Swedish studies investigated the prevalence of CML among first-degree relatives of CML patients utilizing registry data [59, 60]. The first study examined 9491 first-degree relatives of 4619 CML patients diagnosed between 1958 and 2004, and compared them with 42,474 first-degree relatives of matched controls. However, the diagnosis of CML did not incorporate Ph and/or *BCR::ABL1* [59]. Using the same registries which later incorporated Ph and/or *BCR::ABL1*, the second study examined 4,287 first-degree relatives of 88 CML patients diagnosed between 2002 and 2013 and compared them with 20,930 first-degree relatives of matched controls [60]. Both studies showed no increase odds ratio of haematological or solid cancers in the first-degree relatives of CML patients. However, these studies do not report the demographic comparison between the two cohorts and are limited by the nature of the data source and natural history of CML that CML patients could be asymptomatic and undiagnosed for a long period of time. There was no confirmative conclusion could be made from the above non-case report studies in view of the study limitations.

If there is no increased risk of getting CML in relatives of CML patients, it does not eliminate the possibility of familial influence in CML occurrence. To our knowledge, there was only one study [61] explored the familial influence in CML occurrence by comparing the prevalence of harbouring *BCR::ABL1* in first-degree relatives of CML patients and control group. The study performed qualitative PCR to detect *BCR::ABL1* M-BCR in 46 normal first-degree relatives from nine CML families and 52 parents/siblings from 10 families without CML. The M-BCR positivity rate in the relative and control group was 33% and 25%, respectively, though not statistically significant. This study is limited by small sample size and demographic differences between the two groups. The male-to-female ratio was 0.53 versus 0.67 and mean age was 28.6 versus 36.5 in the relative and control group, respectively. CML is a relatively uncommon haematological cancer, primarily affecting male aged 60 years old and above [46].

Previously, we conducted a study to determine the prevalence of harbouring *BCR::ABL1* M-BCR in our local adult normal population (designated as Study^N^ subsequently in this manuscript) [31]. In this manuscript, we present our current study to determine the prevalence of harbouring *BCR::ABL1* M-BCR in first-degree relatives of local CML patients using *BCR::ABL1*-qPCR^IS^ (designated as Study^R^ subsequently in this manuscript). We compared and discussed the prevalence of Study^R^ and Study^N^ [31] to assess the familial influence on CML occurrence.

## Methods

### Ethic and informed consent

Study^R^ received approval from the Ethic Committee of Faculty of Medicine and Health Sciences, Universiti Malaysia Sarawak (UNIMAS) (ref no: UNIMAS/TNC(PI)/09–65/01 [3]). It adhered to the Malaysian Good Clinical Practice Guideline, aligning with the ethical principles outlined in the Declaration of Helsinki. Written informed consent was obtained from all participating relatives.

### Study design

Study^R^ was a cross-sectional study using convenience sampling. Local CML patients followed-up under government hospitals in southern and middle zone of Sarawak were approached. After getting their verbal consent, their first-degree relatives (parents, siblings, including half-siblings, or offspring) were approached. Inclusion criteria were aged 18 years old or above, capable of providing consent, and able to attend scheduled blood taking session at stations in southern and middle zone of Sarawak. Exclusion criteria were refusal to participate and a history of haematological tumour. Demographic data e.g., age, gender, ethnic, weight, height, alcohol consumption, smoking habit, etc., was obtained. About 15 mL of peripheral blood was collected for FBC and *BCR::ABL1*–qPCR^IS^ testing. Primary end point was to study the prevalence of positive *BCR::ABL1*–qPCR^IS^ among first-degree relatives of CML patients.

### Laboratory methodology

Laboratory methodology was similar to Study^N^ [31]: FBC was performed using an automated haematology analyser (XS800i, Sysmex). Total RNA was extracted using TRIzol® according to manufacturer’s protocol after red blood cell lysis. The RNA extracted underwent one-step RT-PCR and the *BCR::ABL1*–qPCR^IS^ test using the SuperScript™ III Platinum™ One-Step qRT-PCR Kit (Invitrogen), the RNaseOUT™ Recombinant Ribonuclease Inhibitor (Invitrogen), and MolecularMD BCR–ABL1^IS^ MR3 Assay™ according to the manufacturers’ protocols. The MolecularMD kit contains a *BCR::ABL1* primer, *ABL1* primer, three controls [negative, low (0.1%^IS^), and high (10%^IS^)], six calibrators [3e5 (300,000 copies/10 µL), 3e4 (30,000 copies/10 µL), 3e3 (3000 copies/10 µL), 3e2 (300 copies/10 µL), 3e1 (30 copies/10 µL), and 3e0 (3 copies/10 µL)] and nuclease-free water. Fusion gene (*BCR::ABL1*) and control gene (*ABL1*) transcripts were amplified in at least two replicates for each sample. The percentage of the *BCR::ABL1*:*ABL1* copy number ratio was multiplied by a correction factor of 0.98, as specified in the MolecularMD kit, to obtain the percentage in IS [63].

The differences between Study^R^ and Study^N^ were the qPCR machine and testing site. Study^N^ used LightCycler 96 at Kumamoto University, while Study^R^ used Roche LightCycler 480II at UNIMAS. The software program used for quantification cycle (Cq) calling in Study^R^ was the Second Derivative Maximum Method.

**Fig. 1:**
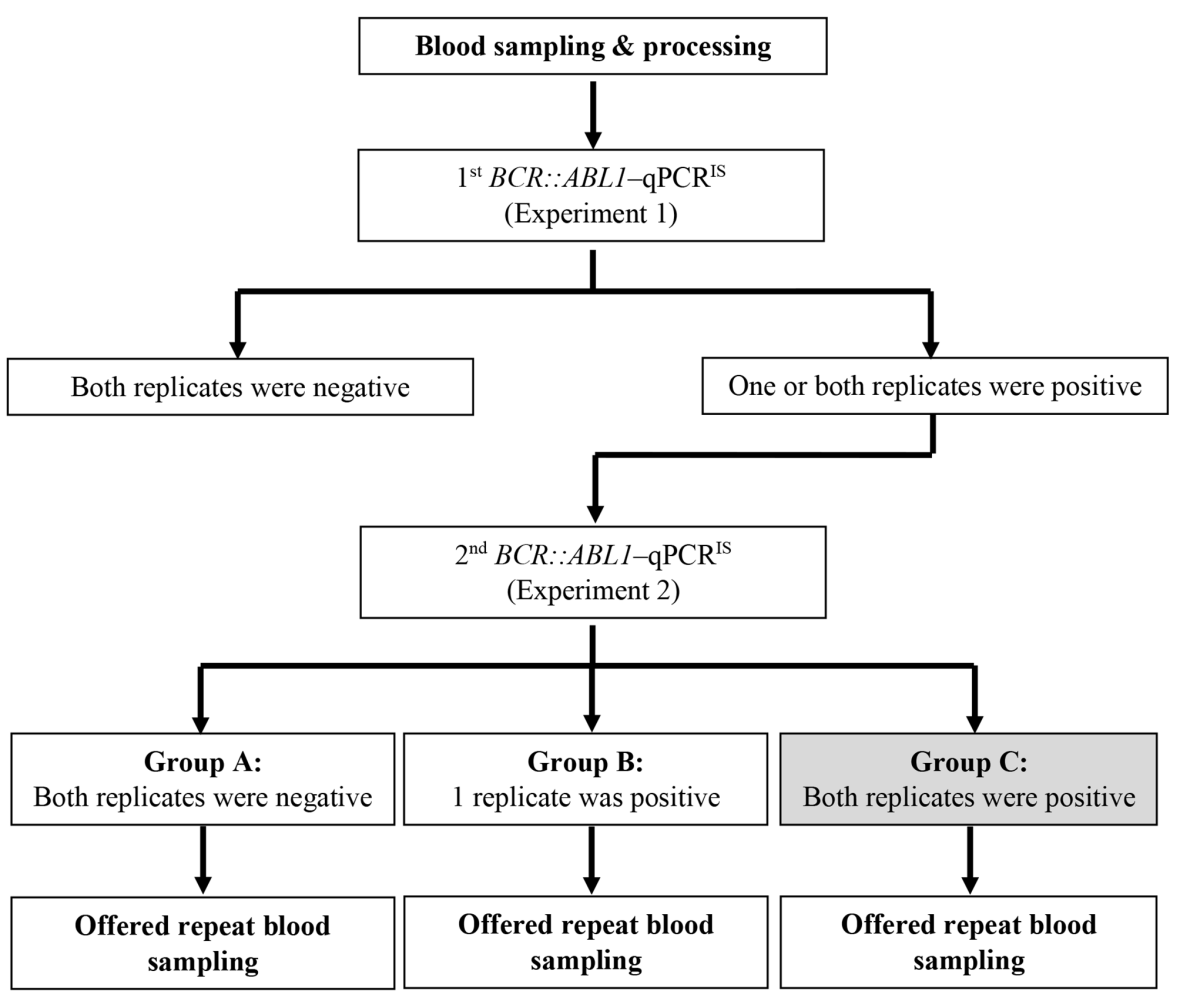
Flow chart of the study. Group C was defined as harbouring *BCR::ABL1* by the study operational definition

Figure 1 illustrates the *BCR::ABL1*–qPCR^IS^ workflow for both Study^R^ and Study^N^. If one or both replicates in the first experiment (Experiment 1) were positive, a repeat experiment of the same sample (Experiment 2) was performed. If both replicates in Experiment 2 were positive, the relative was determined positive for harbouring *BCR::ABL1*. If one or both replicates tested positive in Experiment 1, the option of repeated blood sampling and testing (follow-up) was offered, contingent upon consent.

The laboratory procedure was conducted cautiously to avoid contamination e.g., usage of biosafety/laminated flow cabinet and different workstation/laboratory for lysis, RNA extraction, and *BCR::ABL1*–qPCR^IS^. Each *BCR::ABL1*–qPCR^IS^ included negative controls for *BCR::ABL1* and nuclease-free water for *BCR::ABL1* and *ABL1*. Besides that, there were other *BCR::ABL1*–qPCR^IS^ evaluation criteria used to exclude possibility of contamination.

### Statistical analysis

Data was entered into Microsoft® Excel® for Microsoft 365 MSO. Descriptive statistics was analysed using the same software. The data (relative cohort) was compared to the previous study on the normal population (population cohort) [31] using RStudio (version 2022.12.0 + 353 “Elsbeth Geranium” Release (7d165dcfc1b6d300eb247738db2c7076234f6ef0, 2022-12-03) for Windows Mozilla/5.0 (Windows NT 10.0; Win64; x64)). Chi-square (χ2) test for categorical data and analysis of variance (ANOVA) or Kruskal–Wallis test for normally and non-normally distributed continuous data, respectively, were used to compare the two cohorts. The significant level was set at 0.05, unless otherwise specified.

## Results

A total of 103 first-degree relatives participated and had *BCR::ABL1*–qPCR^IS^ done between February and May 2022. Seven relatives were removed from analysis because there was no written data form. Data of the 96 relatives are presented below.

The 96 relatives were from 41 families. Among them, 18 (19%) were parents, 39 (41%) were siblings, and 39 (41%) were offspring of CML patients. Two of the siblings were half-siblings who shared one biological parent, while the rest were full siblings with the same biological parents. The median number of first-degree relatives per family enrolled was 2 (range 1 to 5).

The demographic characteristics of the relatives are shown in Table 1. The mean age was 39 years old, but the age distribution was skewed to left and closely resembled the Sarawak population [47, 64] (Supplementary Fig. 1). There were more female (53%) and a higher proportion of individuals of Chinese ethnicity (46%). The majority of them were from southern zone (83%) and were overweight or obese (70%), but did not report presence of comorbidity (71%), past history of tumour (99%), family history of non-CML tumour (79%), history of smoking (70%), and did not consume alcohol or only consume it during festival/confinement (83%). As expected, the mean TWC, basophil count and eosinophil count were within normal range. The RNA quality was indicated by the sum of copy number (SCN) of the control gene, *ABL1*. 60% of the samples exhibited good quality, with SCN^*ABL1*^ of 100,000 or more, meeting the criteria for evaluating a 5-log reduction in molecular response (MR5) or deeper when assessing CML patients on treatment.

**Table 1:** The demographic of the first-degree relatives of known chronic myeloid leukaemia patients studied in the current study (Study^R^) and normal population studied in our previous study (Study^N^)

Characteristics	Study^R^ (*n* = 96)	Study^Na^ (*n* = 190)	Sarawak population
**Age (years)**	39 [14]	45 [13]	
**Age group (years), n (%)**			
18–29	29 [30]	24 [13]	695,700 [27]^b, c^
30–39	23 [24]	36 [19]	331,100 [13]^b^
40–49	18 [19]	66 [35]	309,100 [12]^b^
50–59	18 [19]	34 [18]	251,100 [10]^b^
60–69	6 (6.3)	25 [13]	156,100 [6]^b^
70–79	2 (2.1)	3 (1.6)	107,300 [4]^b^
80 or more	0 (0)	2 (1.1)	
**Gender, n (%)**			
Female	51 [53]	112 [59]	1,242,700 [49]^b^
Male	45 [47]	78 [41]	1,289,400 [51]^b^
**Ethnic, n (%)**			
Chinese	44 [46]	51 [27]	611,900 [24]^b^
Dayak	35 [36]	73 [38]	1,193,500 [47]^b, d^
Malay	17 [18]	62 [33]	616,400 [24]^b^
Indian	0 (0)	1 (0.5)	110,300 [4]^b^
Others	0 (0)	3 (1.6)	
**Residential area in Sarawak, n (%)**			
Southern zone^e^	80 (83)	190 (100)	1,274,600 [46]^e^
Middle zone^e^	16 [17]	0 (0)	968,200 [35]^e^
**Comorbid, n (%)**			
Nil /not reported	62 [65]	94 [49]	
Yes	34 [35]	96 [51]	
**Past history of tumour, n (%)** ^**f**^			
No	95 (99)	173 (91)	
Yes, benign or under investigation	0 (0)	15 [8]	
Yes, malignant	1 [1]	2 [1]	
**Family history of tumour, n (%)** ^**g**^			
No	74 (77)	117 [62]	
Yes	22 [23]	73 [38]	
**Smoking, n (%)**			
No	67 (70)	131 (69)	
Quit	8 [8]	21 [11]	
Active	21 [22]	38 [20]	
**Alcohol consumption, n (%)**			
No or only during festival/ confinement	81 (84)	165 (87)	
Quit	4 [4]	9 (4.7)	
Active	11 [11]	16 (8.4)	
**BMI (kg/m**^**2**^)	26.2 (5.8)	27.5 (17.8)	
**BMI status, n (%)**			
Normal	28 [29]	48 [25]	
Overweight	33 [34]	66 [35]	
Obese	35 [36]	76 [40]	
**Total leucocyte count (x 10**^**9**^/L)	7.05 (2.18)	7.44 (2.04)	
**Basophil count (x 10**^**9**^/L)	0.04 (0.04)	0.09 (0.23)	
Unknown, n	1	4	
**Eosinophil count (x 10**^**9**^/L)	0.29 (0.22)	0.29 (0.25)	
Unknown, n	1	0	
**SCN** ^***ABL1***^ **in Experiment 1**	119,009 (67,607)	91,941 (62,412)	
**SCN** ^***ABL1***^ **in Experiment 1, n (%)**			
Less than 20,000	0 (0)	43 [23]	
20,000 to 31,999	1 [1]	10 [5]	
32,000 to 99,999	38 [40]	35 [18]	
100,000 or more	57 [60]	102 [54]	

The values were in mean (standard deviation) unless specified otherwise. BMI, body mass index; SCN, sum of copy numbers in both replicates

^a^the data was taken from our previous study of normal population in southern Sarawak [31]

^b^based on whole Sarawak citizen population in 2015 [47]

^c^age 15 to 29 years old

^d^consists of three Dayak (Sarawak native) ethnic groups only i.e., Iban, Bidayuh, and Melanau

^e^based on whole Sarawak population in 2016 [64]; southern zone consists of the following districts: Kuching, Samarahan, Sri Aman, and Betong; middle zone consists of the following districts: Sarikei, Sibu, Mukah, Kapit and Bintulu

^f^there was no relative with history of haematological tumour because it was in the Study^R^ exclusion criteria

^g^xcluding CML or no family history of CML

Out of the 96 relatives, 16 tested positive for *BCR::ABL1* in one or both replicates during Experiment 1. In Experiment 2, eight out of the 16 tested negative for *BCR::ABL1* in both replicates (Group A), five tested positivity in one replicate (Group B), and four tested positive in both replicates (Group C) (Table 2).

**Table 2:** Sum of *ABL1* copy number, difference of *BCR::ABL1* copy number in both replicates and *BCR::ABL1*-qPCR^IS^ of 16 samples that were positive in Experiment 1 in the current study (Study^R^)

Result in Experiment 2		*N*	Experiment 1			Experiment 2		
			SCN	*BCR::ABL1* difference^a^	qPCR^IS^ (%^IS^)	SCN	*BCR::ABL1* difference^a^	qPCR^IS^ (%^IS^)
**Group A**	Negative in both replicates	8	101,346 (42,877, 164,165)	2.0 (1.3, 3.7)	0.0034 (0.0010, 0.0069)	198,082 (125,160, 345,054)	0	0
**Group B**	Positive in 1 replicate	5	238,109 (172,285, 376,521)	0.8 (0, 1.7)	0.0025 (0.0008, 0.0034)	218,412 (113,906, 283,780)	7.3 (2.9, 18.5)	0.0035 (0.0010, 0.0091)
**Group C**	Positive in both replicates	4^**b**^	210,905 (172,121, 256,736)	4.2 (1.5, 6)	0.0043 (0.0017, 0.0071)	100,922 (45,344, 174,008)	8.0 (0.7, 19.5)	0.0327 (0.0055, 0.0934)

Result was in mean (minimum, maximum). SCN, sum of copy numbers in both replicates

^a^difference of copy numbers in both replicates

^b^these 4 relatives are designated as R1, R2, R3, and R4 in the other related tables and text

According to the study operational definition, the four (4.2%) relatives in Group C (designated as R1, R2, R3, and R4) harboured *BCR::ABL1* (Table 3). Notably, R2 and R3 were from the same family. There was no repeat blood sampling for R1 to R4 due to various logistical reasons. In September 2022, repeat blood sampling was conducted for three individuals in Group A and two individuals in Group B. All of them tested negative in both replicates of Experiment 1, with a mean SCN^*ABL1*^ of 115,364.

**Table 3:** Demography of the relatives who were determined as having a positive *BCR::ABL1*-qPCR^IS^ result in the current study (Study^R^)

No.	ID	Age	Gender	Ethnic	Residential area in Sarawak	Relationship to index case
**1**	R1	31	Female	Chinese	Middle zone	Sibling
**2**	R2	30	Female	Chinese	Middle zone	Sibling
**3**	R3	55	Female	Chinese	Middle zone	Parent
**4**	R4	18	Male	Chinese	Middle zone	Sibling

Although there were more female than male relatives harbouring *BCR::ABL1*, and all positive relatives are Chinese, the differences were not statistically significant, as well as the other characteristics (Table 4). The characteristics that were significant were the residential zone being in the middle zone and a higher SCN^*ABL1*^.

**Table 4:** The association between sociodemographic characteristics with the presence of *BCR::ABL1* in the current study (Study^R^)

Characteristics	Number of relatives harbouring *BCR::ABL1*, n (%)		p-value
	Yes (*n* = 4)	No (*n* = 92)	
**Age (years)**	33 [16]	39 [14]	0.497
**Gender**			0.701
Male	1 [25]	44 [48]	
Female	3 (75)	48 [52]	
**Ethnicity**			0.088
Chinese	4 (100)	40 [43]	
Non-Chinese	0 (0)	52 [57]	
**Residential zone**			< 0.001
Middle zone	4 (100)	12 [13]	
Southern zone	0 (0)	80 (87)	
**Relationship to index case**			0.281
Sibling	3 (75)	40 [43]	
Parent	1 [25]	16 [17]	
Offspring	0 (0)	36 (39	
**Co-morbid**			> 0.999
Yes	1 [25]	33 [36]	
No	3 (75)	59 [64]	
**Family history of tumours**			0.613
Yes	0 (0)	22 [24]	
No	4 (100)	70 (76)	
**Past history of tumours**			> 0.999
Yes	0 (0)	1 [1]	
No	4 (100)	91 (99)	
**Smoking, n (%)**			0.405
No	63 (68)	4 (100)	
Quit	8 [9]	0 (0)	
Active	21 [23]	0 (0)	
**Alcohol consumption, n (%)**			0.679
No or only during festival/ confinement	77 (84)	4 (100)	
Quit	4 [4]	0 (0)	
Active	11 [12]	0 (0)	
**BMI (kg/m**^**2**^)	24.9 (5.4)	26.2 (5.8)	0.673
**Total leucocyte count (x 10**^**9**^/L)	5.45 (0.57)	7.12 (2.20)	0.002
**Basophil count (x 10**^**9**^/L)	0.09 (0.09)	0.04 (0.03)	0.375
Unknown, n	0	1	
**Eosinophil count (x 10** ^**9**^ **/L)**	0.29 (0.21)	0.29 (0.22)	0.992
Unknown, n	0	1	
**SCN** ^**ABL1**^ **in Experiment 1**	210,905 (42,482)	115,014 (65,763)	0.015

The values were in mean (standard deviation) unless specified otherwise. BMI, body mass index; SCN, sum of copy numbers in both replicates

## Discussion

### Higher prevalence of harbouring *BCR::ABL1* M-BCR in first-degree relatives of CML patients (current study, Study^R^) compared to normal population (our previous study, Study^N^)

Previously, we conducted a study, Study^N^, to determine the prevalence of harbouring *BCR::ABL1* M-BCR in our local adult normal population, using an unbiased sampling method [31]. The sampling method employed a two-stage sampling approach based on the Malaysia Department of Statistics population survey procedure. The first- and second-stage samplings were stratified sampling (selection of enumeration blocks (EB) based on the population density of Kuching Division and Samarahan Division) and cluster sampling (selection of 12 living quarters (LQ) out of all LQs in each EB), respectively. Study^N^ should be viewed and served as a baseline data.

In our current study, Study^R^, we found that four (4.2%) (designated as R1 to R4) out of 96 first-degree relatives of CML patients harboured *BCR::ABL1* M-BCR, whereas Study^N^ showed that one (0.5%) (designated as P1) out of 190 subjects in normal population harboured *BCR::ABL1* M-BCR [31]. When considering samples with SCN^*ABL1*^ ≥ 100,000, Study^R^ and Study^N^ revealed that four (7%) out of 57 and one (1%) out of 102 subjects were positive, respectively. These findings suggest a higher prevalence of harbouring *BCR::ABL1* in first-degree relatives of CML patients compared to the normal population.

The demographic differences between these two studies (Table 1) initially seem to hinder further comparison. However, further interpretation heightens the significance of the higher prevalence in the relative cohort, as elaborated below.

The mean age in Study^R^ was 39, compared to 45 in Study^N^. The mean age of R1 to R4 was 33.5, compared to 44 for P1. The age difference between Study^R^ and Study^N^ heightens the significance of the higher prevalence in relative cohort because CML is more common in older age group of 55 and above [65]. Several studies on normal subjects harbouring *BCR::ABL1* also showed a higher prevalence in older age groups [20, 25, 27]. The younger age in Study^R^ corresponds to the younger mean age of diagnosis in the case reports of familial CML, which is 46 (median 49, range 0.8 to 73) (Supplementary Table 3).

The prevalences of harbouring *BCR::ABL1* M-BCR in Study^R^ were 5.5% and 2.1% for female and male relatives, respectively, while in Study^N^ it was 1.3% for male normal population. This finding is inconsistent with existing knowledge, as CML is known to be male predominant globally, with a male-to-female ASIR (ASIR-M:F) ratio of about 1.2 to 1.3 between 1990 and 2017 [46]. Interestingly, Meza-Espinoza JP et al. [61] also showed a female predominance in their study. The positive rate of female and male was 40% and 18.8%, respectively, in the relative group, and 31.3% and 15%, respectively, in the control group. Thus, there is a possibility that familial influence in CML occurrence is more pronounced in female than in male.

The positive relatives in Study^R^ were all Chinese, while the positive subject in Study^N^ was Malay. This finding is inconsistent with our local CML epidemiology. The ASIR of adult CML in Sarawak was higher in Malay, 0.7 per 100,000 population, compared to Chinese, 0.4, from 1996 to 2015 [47]. This might suggest a stronger familial influence in CML occurrence among Chinese individuals, while a stronger non-familial risk factor may be present and causing a higher occurrence of CML in Malay.

### *BCR::ABL1*–qPCR^IS^ level

Considering data from Experiment 1, the *BCR::ABL1*–qPCR^IS^ level in Study^R^ and Study^N^ were similar, ranging from 0.0017%^IS^ to 0.0071%^IS^ in Study^R^ and 0.0023%IS to 0.0032%IS in Study^N^ [31]. The lowest detectable *BCR::ABL1* level in standard clinical haematological practice is around MR4 (0.01%^IS^), MR4.5 (0.0032%^IS^), and MR5 (0.001%^IS^), indicating a good response in CML patients receiving treatment [66]. This finding supports the argument against employing more expensive *BCR::ABL1*–qPCR^IS^ test detecting response deeper than MR4.5 in clinical practice.

Besides Study^R^ and Study^N^, to our knowledge, only Fenu E et al. have performed *BCR::ABL1*–qPCR^IS^. The levels ranged between 0.006%^IS^ and 0.016% ^IS^ in four subjects [30], which is similar to Study^R^ and Study^N^. Only two of them were re-tested after 10 days and three months, respectively, and the results were negative [30]. 

The higher mean SCN^*ABL1*^ of the four positive relatives compared to the negative relatives in Study^R^ (*p* = 0.015) was within expectation. A good quality sample is required to detect a low level of *BCR::ABL1* in normal population.

### Study limitation

The main study limitation of Study^R^ is the small sample size. The lack of statistical significance in the association between female gender and Chinese ethnicity in the positive relatives may be attributed to this small sample size. Conducting a future study with similar demographics between a relative and normal cohort is ideal, but logistical challenges may arise. Using the results of Study^R^ and Study^N^, along with OpenEpi software with a two-sided confidence level (1-alpha) of 95, 80% power, a 1:1 ratio of controls to cases, and hypothetical exposure proportions of 0.5 for controls and 4 for cases, the sample size needed for an unmatched case-control study was estimated to be about 280 in each case and control group.

Residential zone differed between Study^R^ and Study^N^. R1 to R4 resided in the middle zone of Sarawak, as did three and all subjects in Group A and B, respectively. Study^N^ was conducted only in the southern zone [31], making it uncertain how residential location might influence our findings. We recommend a study investigating the prevalence of harbouring *BCR::ABL1* in the normal population within the middle zone. Notably, the incidence rate of CML in southern and middle zone of Sarawak between 1996 and 2015 was 0.5 and 0.4 per 100,000 population, respectively (unpublished data in Kuan JW et al. [47]).

Repeat blood sampling is ideal for rectification and follow-up in positive cases, but unfortunately, it wasn’t possible for participants R1 to R4 due to logistical constraints. Repeat blood sampling was done for P1 in Study^N^ five months after detecting *BCR::ABL1* and the result was negative with SCN^*ABL1*^ of 232,077 [31]. 

The familial influence in CML occurrence might be diluted, for example in offspring, because of the presence of the other parent’s genetic features. In Study^R^, three (7.7%) out of 39 siblings and one (5.5%) out of 18 parents were positive. None of 39 offspring was positive. Further research on the “dilutional genetic effect” in larger studies would be of interest. Notably, protective genetic factors are influential, as evidenced by immunotherapy like interferon in CML treatment. Another evidence comes from CML treatment-free remission studies, where NK-cell-based immune surveillance may contribute to CML control after TKI cessation.

## Conclusion

Our study showed that the prevalence of harbouring *BCR::ABL1* in the first-degree relatives of known CML patients was approximately 4%, higher than the 0.5% prevalence observed in the normal population. This suggests that familial influence in CML occurrence might exist but could be surpassed by other more dominant influences, such as genetic dilutional effects and protective genetic factors. Further investigation into this topic is warranted, ideally through larger studies with longer follow-up periods.

### Electronic supplementary material

Below is the link to the electronic supplementary material.

Supplementary Material 1

## Data Availability

The data that support the findings of this study are available from UNIMAS but restrictions apply to the availability of these data, which were used under license for the current study, and so are not publicly available. Data are however available from the authors (Prof Dr Kuan Jew Win) upon reasonable request and with permission of UNIMAS.

## Abbreviations

AP: acceleration phase of CML
ASIR: Age-standardised Incidence Rate
BC: blast crisis of CML
BMA: bone marrow aspiration
CML: chronic myeloid leukaemia
CP: chronic phase of CML
FBC: full blood count
IS: International Scale
M-BCR: major transcripts of *BCR::ABL1*, namely e13a2 and e14a2
MR: molecular response in the quantity unit of log reduction
M:F: male-to-female
PBF: peripheral blood film
PCR: polymerase chain reaction
Ph: Philadelphia chromosome
pre-CP: pre-clinical CML or pre-clinical phase of CML
qPCR^IS^: IS-standardised real-time quantitative polymerase chain reaction
SCN: sum of copy number
TKI: tyrosine kinase inhibitor
TWC: total white cell count
UNIMAS: Universiti Malaysia Sarawak
