# Supplementary material for: Higher prevalence of harbouring BCR::ABL1 in first-degree relatives of chronic myeloid leukaemia (CML) patients compared to normal population
Source: BMC Cancer. 2024 Jun 14;24:734. doi: 10.1186/s12885-024-12102-2 (PMC11179337; doi:10.1186/s12885-024-12102-2)
Supplement: Supplementary file 1 — Supplementary Material 1 [file 12885_2024_12102_MOESM1_ESM.pdf]

Supplementary Data

Contents

Supplementary Table 1 2
Supplementary Table 2 5
Supplementary Table 3 6
Supplementary Figure 1 8
REFERENCES 9

Supplementary Table 1

We used our previous criteria(1) to include or exclude the studies into this table.

- The inclusion criterion was total white blood cell (TWC) ≤ 15 × 10^9/L.
- The exclusion criteria were:
  (1) No TWC or cytogenetics/PCR data,
  (2) CML variant presented with normal TWC and only thrombocytosis with Ph chromosome and/or *BCR::ABL1* fusion gene positive(2) (this variant was previously thought to be Essential Thrombocythaemia), and
  (3) Post-allogenic haematopoietic stem cell transplantation (HSCT) (to eliminate potential treatment or immunomodulating effect from allogenic HSCT).

We also used our previous criteria(1) to determine the time point of CML CP diagnosis:

- TWC > 15 × 10^9/L in the presence of typical peripheral blood film (PBF) and bone marrow aspiration (BMA) features(2), along with the presence of Ph and/or *BCR::ABL1*(2).
- If TWC ≤ 15 × 10^9/L, the diagnosis required absolute basophilia (absolute basophil count ≥ 0.2×10^9/L), typical PBF and BMA features, and Ph in ≥ 75% of the examined metaphases.
- Once CML CP was diagnosed using above criteria, subsequent data was excluded even if the laboratory data did not meet the CP criteria mentioned above.

Supplementary Table 1. Summary of the selected case reports on pre-clinical CML with available parameters of interest

No.	Study	Group[g]	Age (year)	Gender	TWC (x 10^9/L)	Hb (g/dL)	Platelet (x 10^9/L)	Eosinophil (x 10^9/L)	Basophil (x 10^9/L)	%Ph in cytogenetic	Duration (months)[e]
1.	Canellos GP 1972(3)	3	43	M	8.8	15.8	181	NR	NR	10	16
2.	Berman E 1991(4)	1	32	M	6.6	14.8	386	NR	NR	14.3	17
3.	Hudnall SD 2007(5)	2	33	M	9.3	11.7	440	0.2	0.1	70	16
					NR	NR	NR	NR	NR	38.4	
					6.7	14.1	273	0.4	0.2	70	

4.	Shani D 2009(6)	2	74	M	3	8.25	165	NR	NR	15[a]	NR
5.	Bayraktar S 2010(7)	1	39	M	15	15	308	NR	NR	17[b] (PB)	12
					7.6	15.3	364	NR	0.4	20[c]	
6.	Roper N 2010(8)	1	60	M	7.3	13.1	138	NR	NR	75	17
7.	Aye LL 2016(9)	2	70	M	3.6	11.4	225	0	0.1	35	NR
8.		3	70	M	14.7	13.4	412	0.1	0	15	
9.		2	67	M	12.7	8.4	395	0.6	0.1	15	
10.	Morita K 2016(10)	1	64	F	5.6	12.6	274	normal	normal	20	48
11.	Alsidawai S 2014(11)	1	60	M	11.2	9.5	509	mild ↑	normal	53	3
12.	Abecasis M 2020(12)	3	0	F	13	9.4	215	NR	NR	50[d]	6
13.	Rivera D 2024(13)	1	63	F	9.3	8	187	0.2	0.2	45	NR
14.		1	49	F	11.1	14.3	351	0.2	NR	8.0[b] (PB)	NR
15.		1	70	M	7.6	16.5	132	0.0	0.1	30	NR
16.		1	73	F	2.9	8.3	109	NR	NR	40	NR
17.		1	35	F	6.5	13.7	199	0.1	0.1	40	NR
18.		1	56	F	2.9	11.7	123	0.2	0.0	15	NR
19.		2	78	F	8.4	11.5	146	0.3	0.2	30	NR
20.		2	50	F	10.5	9.8	260	0.1	0.2	63	NR
21.		2	55	F	7.7	12.8	277	0.3	0.1	45	NR
22.		2	65	M	11	14.6	206	0.2	0.1	60	NR
23.		2	65	M	3.5	8.9	136	0.0	0.0	24[b] (BM)	NR
24.		2	70	F	26.4	12.5	36	0.3	NR	45	NR
25.		2	50	M	8.7	11.7	370	0.1	NR	30[b] (BM)	NR
26.		3	64	M	9.1	16.8	456	0.4	0.5	70[b] (PB)	NR
27.		3	44	F	3.6	11.4	225	NR	0.1	20	NR

28.		3	80	F	7.6	12.5	199	0.9	0.1	33	NR
29.		3	79	F	10.3	9.3	443	0.6	0.6	50	NR
30.		3	66	M	8.4	12.7	395	0.1	NR	18	26
**Median (min, max)**			**63.5 (0, 80)**	**M 53% F 47%**	**8.4 (2.9, 26.4)**	**12.5 (8, 16.8)**	**242.5 (36, 509)**	**0.2 (0, 0.9)**	**0.1 (0, 0.6)**	**35 (10, 75)**	**16 (3, 48)**

CML, chronic myeloid leukaemia; CP, chronic phase; F, female; Hb, haemoglobin; M, male; NR, not reported/done/available; Ph, Philadelphia chromosome; TWC, total white blood cell.

[a]the study only reported Ph was detected in 3 metaphases. Assumed total of 20 metaphases were analysed.

[b]result from Fluorescence In-Situ Hybridization (FISH). The sample source was indicated as peripheral blood (PB) or bone marrow (BM).

[C]result was from BM that showed Ph in 4 of 20 metaphases. FISH was positive in 52% of the BM cells.

[d]result from retrospective analysis of the patient's cord blood sample using nested genomic DNA-based PCR of which *BCR::ABL1* fusion was identified in 2/4 replicates. The baby girl was diagnosed with CML CP at the age of 6 months.

[e]from the first evidence of pre-clinical CML till diagnosis of CML CP (see text for criteria of CML CP).

[g]group 1 indicates patients who had a history of cytotoxic therapy ± radiotherapy for hematopoietic or nonhematopoietic malignancies.
group 2 indicates patients who had prior or concurrent hematopoietic or nonhematopoietic malignancies but did not receive cytotoxic therapy ± radiotherapy.
group 3 indicates patients without prior or concurrent malignancies.

[h]result was derived solely from cytogenetic result.

Note:

1. Search strategy and term: see Kuan JW *et al*(1).
2. May not be a complete list because last search was July 2017. Subsequent studies were found based on web notifications.

Supplementary Table 2

Supplementary Table 2. Prevalence of *BCR::ABL1* M-BCR positivity in normal subjects/population

No.	Study	CB/NB/I	Children	Adult	Total
1.	Biernaux C 1995(14)	0/22 (0)	1/22 (4.5)	22/73 (30.1)	23/117 (19.7)
2.	Van Rhee F 1996(15)	-	-	0/20 (0)	0/20 (0)
3.	Bose S 1998(16)	-	-	4/15 (26.7)	4/15 (26.7)
4.	Ravetto PF 2003(17)	0/88 (0)	-	-	0/88 (0)
5.	Hsu H 2004(18)	-	-	1/51 (2)	1/51 (2)
6.	Winn-Deen ES 2007(19)	-	-	0/44 (0)[c]	0/44 (0)
7.	le Coutre P 2010(20)	-	-	5/100 (5)	5/100 (5)
8.	Song J 2011(21)	10/60 (16.7)	9/24 (37.5)	23/40 (57.5)	42/124 (33.9)
9.	Boquett JA 2013(22)	-	-	2/30 (6.7)	2/30 (6.7)
10.	Ismail SI 2014(23)	-	4/44 (9.1)	15/145 (10.3)	19/189 (10.1)
11.	Meza-Espinoza JP 2019(24)[a]	-	-	28/98 (28.6)	28/98 (28.6)
12.	Kuan JW 2020(25)	-	-	1/190 (0.5)	1/190 (0.5)
13.	Fenu E 2021(26)[b]	-	-	4/471 (0.8)	4/471 (0.8)
14.	Current study[a]	-	-	4/103 (3.9)	4/103 (3.9)
**Total**		**10/170 (5.9)**	**14/90 (15.6)**	**109/1380 (7.9)**	**133/1640 (8.1)**

CB/NB/I, cord blood/newborn/infant. Result in n/N (%).

[a]study includes relatives of known CML patients.

[b]study on patients with peripheral blood cytoses but were not diagnosed as CML later.

[c]assumed adult.

Note:

1. Search strategy and term: see Kuan JW *et al*(27).
2. May not be a complete list because last search was July 2017. Subsequent studies were found based on web notifications.

Supplementary Table 3

Supplementary Table 3. Summary of the case reports on familial CML

No.	Study	Index case – age & gender	Family members		Duration
			Relationship	Age & gender	
**1.**	Tokuhata GK 1968(28)	64M$^{s+, c+, p0}$	Identical twin$^{s+, c+, p0}$, older brother$^{s-, c+, p0}$	64M, 68M	3 days, 3 months
**2.**	Gunz FW 1975(29)	NA	Relative but not 1st degree	NA	NA
**3.**	Avilés A 1982(30)	34F$^{s+, c+, p0}$	Younger sister$^{s+, c+, p0}$	35F	8 years
**4.**	Lillicrap DA 1984(31)	14F$^{s+, c-, p0}$	Mother$^{s+, c+, p0}$	54F	12 years
**5.**	Spremolla G 1987(32)	43M$^{s+, c+, p0}$	Older brother$^{s+, c+, p0}$	56M	10 years
**6.**	Horwitz M 1996(33)	Quoted 2 families recoded in Videbzek A (1947)(34). 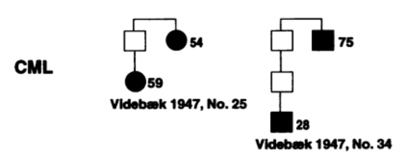			
**7.**	Kapsali E 2000(35)	47M $^{s+, c+, p0}$	Older sister$^{s-, c-, p+}$	62F	7 years
**8.**	Lessen DS 2005(36)	45M $^{s+, c+, p+}$	Older brother$^{s?, c0, p0}$	25M	26 years
**9.**	Caocci G 2009(37)	45F$^{s+, c+, p+}$	Younger brother$^{s?, c+, p+}$	50M	8 years
**10.**	Malak S 2012(38)	Got 2 families with CML. No further elaboration.			
**11.**	Prasad S 2019(39)	28F$^{s+, c+, p?}$	Son$^{s+, c+, p+}$	10M	8 years
**12.**	Tsumura AM 2021(40)	54F$^{s+, c+, p+, d1}$	Son$^{s+, c+, p+, d1}$	49M	17 years
**13.**	Verrou E 2021(41)	54M$^{s+, c+, p+ (e13a2)}$	Older brother$^{s?, c+, p+ (e1a2)}$	63M	4 years
**14.**	Aitken MJL 2021(42)	24F$^{s+, c?, p+, d2}$	Maternal great aunt$^{s+, c+, p+, d2}$	73F	6 months

Age denotes age at diagnosis (year). Duration denotes duration between diagnosis of index case and the relative. CML, chronic myeloid leukaemia; F, female; M, male; MDS/MPN, myelodysplastic/myeloproliferative neoplasms; NA, not available

^{s}symptoms at diagnosis: + positive, - negative, ? unknown/NA.

^{c}cytogenetic and/or fluorescence in situ hybridization (FISH) for *BCR::ABL1* test: + positive for Ph, - negative for Ph, 0 not done, ? unknown/NA.

^{p}PCR for *BCR::ABL1* test (major transcript or assumed major transcript if did not specify in the report): + positive, - negative, 0 not done, ? unknown/NA.

d1, underlying *ANKRD26*-related familial thrombocytopenia.

d2, significant family history of breast, liver, lung, and stomach cancer.
Note:

1. Primary search: all fields in PubMed on 28th August 2023. Search term: (chronic myeloid leukaemia OR chronic myeloid leukemia OR chronic myelogenous leukaemia OR chronic myelogenous leukaemia OR CML) AND (familial OR hereditary OR inherited OR family OR relative). Secondary search: relevant references in selected studies from the primary search.
2. Excluded familial cases with CML and other diseases and cases without evidence of Ph and/or *BCR::ABL1*.
3. Bondare DK 1985(43) – Language: Russia. Unable to find full article. Abstract in English stated "In one of the families, polycythemia vera (PV) was seen in twin brother and sister, in the other one, chronic myeloleukemia (CML) afflicted both daughter and mother, and in the two remaining families PV and CML afflicted two brothers and mother and daughter, respectively."
4. Human leucocyte antigen (HLA) information was only available in one case report(37) and the result was conflicting – the two affected siblings have HLA-A3 (lower risk) and A11 (higher risk), respectively.

Supplementary Figure 1

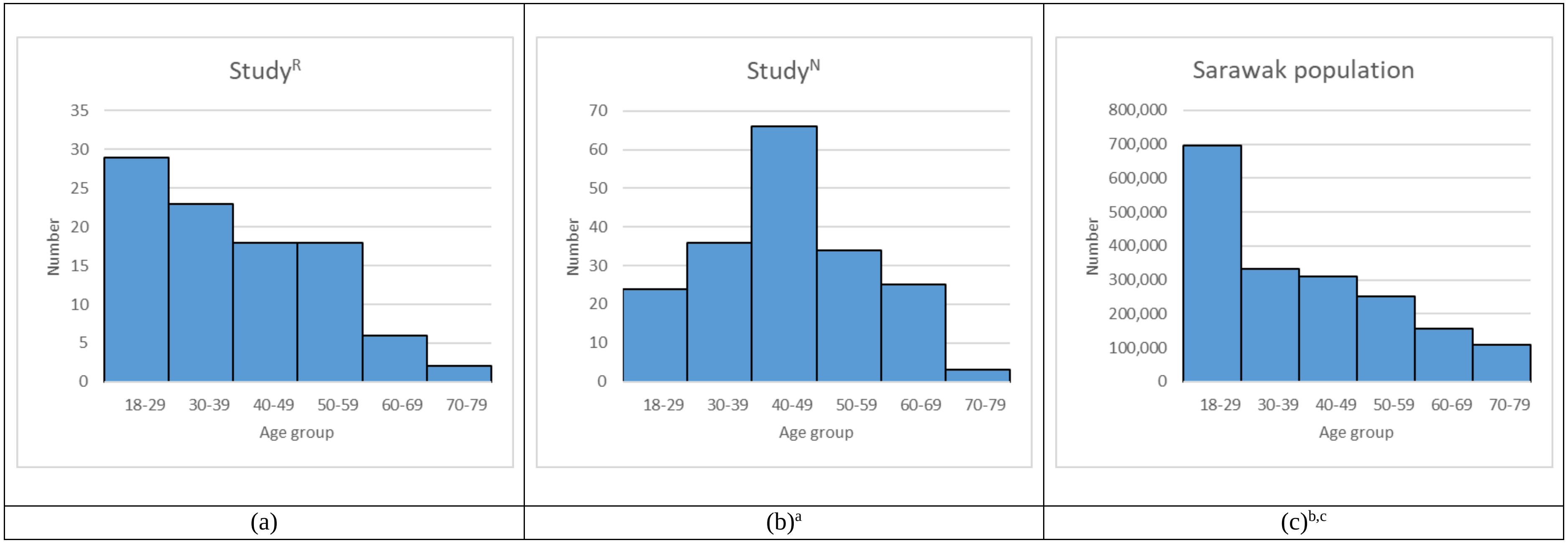

(a)	(b)[a]	(c)[b,c]

Supplementary Figure 1. Distribution according to age group for (a) first-degree relatives of known chronic myeloid leukaemia patients studied in the current study (Study[R]), (b) normal population studied in our previous study (Study[N]), and (c) Sarawak population.

[a]the data was taken from our previous study of normal population in southern Sarawak(25).
[b]based on whole Sarawak citizen population in 2015(44).
[c]the age group 18-29 was actually age 15 to 29 years old.

REFERENCES

1. Kuan JW, Su AT, Leong CF, Osato M, Sashida G. Systematic review of pre-clinical chronic myeloid leukaemia. Int J Hematol. 2018;108(5):465-84.
2. Vardiman JW, Melo JV, Baccarani M, Thiele J. Chronic myeloid leukemia, *BCR-ABL1*-positive. In: Swerdlow SH, Campo E, Harris NL, Jaffe ES, Pileri AS, Stein H, et al., editors. WHO classification of tumors of hematopoietic and lymphoid tissues. Revised 4th ed. Lyon: IARC Press; 2017. p. 32-7.
3. Canellos GP, Whang-Peng J. Philadelphia-chromosome-positive preleukaemic state. Lancet. 1972;2(7789):1227-8.
4. Berman E, Strife A, Wisniewski D, Desai S, Gulati S, Jhanwar S, et al. Duration of the preclinical phase of chronic myelogenous leukemia: a case report. Blood. 1991;78(11):2969-72.
5. Hudnall SD, Northup J, Panova N, Suleman K, Velagaleti G. Prolonged preleukemic phase of chronic myelogenous leukemia. Exp Mol Pathol. 2007;83(3):484-9.
6. Shani D, Malik A. Incidental diagnosis of CML in a patient with anemia and IgG lamda monoclonal protein in blood. Ann Hematol Oncol. 2009;88(10):1041-.
7. Bayraktar S, Goodman M. Detection of BCR-ABL Positive Cells in an Asymptomatic Patient: A Case Report and Literature Review. Case Rep Med. 2010;2010:939706-.
8. Roper N, DeAngelo DJ, Kuo F, Dal Cin P, Ghobrial I, Aster JC. An asymptomatic 61-year-old man with BCR-ABL-positive bone marrow following autologous transplantation for multiple myeloma. Am J Hematol. 2010;85(12):944-6.
9. Aye LL, Loghavi S, Young KH, Siddiqi I, Yin CC, Routbort MJ, et al. Preleukemic phase of chronic myelogenous leukemia: morphologic and immunohistochemical characterization of 7 cases. Ann Diagn Pathol. 2016;21:53-8.
10. Morita K, Nakamura F, Taoka K, Satoh Y, Iizuka H, Masuda A, et al. Incidentally-detected t(9;22)(q34;q11)/BCR-ABL1- positive clone developing into chronic phase chronic myeloid leukaemia after four years of dormancy. Br J Haematol. 2016;174(5):815-7.
11. Alsidawi S, Ghose A, Qualtieri J, Radhakrishnan N. A Case of Multiple Myeloma with Metachronous Chronic Myeloid Leukemia Treated Successfully with Bortezomib, Dexamethasone, and Dasatinib. Case Reports in Oncological Medicine. 2014;2014.
12. Abecasis M, Cross NCP, Brito M, Ferreira I, Sakamoto KM, Hijiya N, et al. Is cancer latency an outdated concept? Lessons from chronic myeloid leukemia. Leukemia. 2020.
13. Rivera D, Cui W, Gao J, Peker D, Zhang Q-Y, Dewar R, et al. Aleukemic Chronic Myeloid Leukemia Without Neutrophilia and Thrombocytosis: a Report From the BCR::ABL1 Pathology Group. Modern Pathology. 2024;37(2).
14. Biernaux C, Loos M, Sels A, Huez G, Stryckmans P. Detection of major bcr-abl gene expression at a very low level in blood cells of some healthy individuals. Blood. 1995;86(8):3118-22.
15. van Rhee F, Hochhaus A, Lin F, Melo JV, Goldman JM, Cross NC. p190 BCR-ABL mRNA is expressed at low levels in p210-positive chronic myeloid and acute lymphoblastic leukemias. Blood. 1996;87(12):5213-7.
16. Bose S, Deininger M, Gora-Tybor J, Goldman JM, Melo JV. The presence of typical and atypical BCR-ABL fusion genes in leukocytes of normal individuals: biologic significance and implications for the assessment of minimal residual disease. Blood. 1998;92(9):3362-7.
17. Ravetto PF, Agarwal R, Chiswick ML, D'Souza SW, Eden OB, Taylor GM. Absence of leukaemic fusion gene transcripts in preterm infants exposed to diagnostic x rays. Archives Of Disease In Childhood Fetal And Neonatal Edition. 2003;88(3):F237-F44.
18. Hsu H, Tan L, Au L, Lee Y, Lieu C, Tsai W, et al. Detection of bcr-abl gene expression at a low level in blood cells of some patients with essential thrombocythemia. Journal of Laboratory & Clinical Medicine. 2004;143(2):125-9.
19. Winn-Deen ES, Helton B, Van Atta R, Wong W, Peralta J, Wang J, et al. Development of an integrated assay for detection of BCR-ABL RNA. Clin Chem. 2007;53(9):1593-600.
20. le Coutre P, Reinke P, Neuhaus R, Trappe R, Ringel F, Lalancette M, et al. BCR-ABL positive cells and chronic myeloid leukemia in immune suppressed organ transplant recipients. Eur J Haematol. 2010;84(1):26-33.

21. Song J, Mercer D, Hu X, Liu H, Li MM. Common leukemia- and lymphoma-associated genetic aberrations in healthy individuals. The Journal Of Molecular Diagnostics: JMD. 2011;13(2):213-9.
22. Boquett JA, Alves JRP, de Oliveira CEC. Analysis of BCR/ABL transcripts in healthy individuals. Genetics And Molecular Research: GMR. 2013;12(4):4967-71.
23. Ismail SI, Naffa RG, Yousef A-MF, Ghanim MT. Incidence of bcr-abl fusion transcripts in healthy individuals. Molecular Medicine Reports. 2014;9(4):1271-6.
24. Meza-Espinoza JP, Vasquez-Jimenez EA, Barajas-Torres RL, Magana-Torres MT, Gonzalez-Garcia JR. BCR/ABL1 Transcripts in Healthy Individuals: A Comparative Analysis Between First-Degree Relatives of Patients with Chronic Myelogenous Leukemia and Subjects without Antecedents of the Disease. Ann Clin Lab Sci. 2019;49(6):703-9.
25. Kuan JW, Su AT, Tay SP, Fong IL, Kubota S, Su'ut L, et al. Low prevalence of the BCR-ABL1 fusion gene in a normal population in southern Sarawak. Int J Hematol. 2020;111(2):217-24.
26. Fenu E, O'Neill SS, Insuasti-Beltran G. BCR-ABL1 p210 screening for chronic myeloid leukemia in patients with peripheral blood cytoses. Int J Lab Hematol. 2021;43(6):1458-64.
27. Kuan JW, Su AT, Leong CF, Osato M, Sashida G. Systematic Review of Normal Subjects Harbouring BCR-ABL1 Fusion Gene. Acta Haematol. 2020;143(2):96-111.
28. Tokuhata GK, Neely CL, Williams DL. Chronic myelocytic leukemia in identical twins and a sibling. Blood. 1968;31(2):216-25.
29. Gunz FW, Gunz JP, Veale AM, Chapman CJ, Houston IB. Familial leukaemia: a study of 909 families. Scand J Haematol. 1975;15(2):117-31.
30. Avilés A, Sinco A, Zapata N, Ambriz R, Pizzuto J. [Familial chronic myelogenous leukemia. Report of 3 cases in a family]. Sangre (Barc). 1982;27(5):953-8.
31. Lillicrap DA, Sterndale H. Familial chronic myeloid leukaemia. Lancet. 1984;2(8404):699.
32. Spremolla G, Simi P, Bilancia D, Papineschi F. [Familial chronic myeloid leukemia. Description of 2 cases in brothers]. Recenti Prog Med. 1987;78(2):69-72.
33. Horwitz M, Goode EL, Jarvik GP. Anticipation in familial leukemia. Am J Hum Genet. 1996;59(5):990-8.
34. Heredity in Human Leukemia and Its Relation to Cancer. Archives of Internal Medicine. 1949;84(3):521-.
35. Kapsali E, Tsiara S, Christou L, Panteli A, Bourantas KL. Two siblings with chronic myelogenous leukemia. J Exp Clin Cancer Res. 2000;19(4):541-3.
36. Lessen DS, Novoselac AV, Hellman G, Tapia A, Ratner LH, Najfeld V. Chronic myelogenous leukemia occurring in two brothers diagnosed 26 years apart. Cancer Genet Cytogenet. 2005;160(1):73-5.
37. Caocci G, Atzeni S, Vacca A, Orrù N, Ledda A, La Nasa G. Familial occurrence of chronic myeloid leukemia. Leuk Lymphoma. 2009;50(5):854-6.
38. Malak S, Labopin M, Saint-Martin C, Bellanne-Chantelot C, Najman A. Long term follow up of 93 families with myeloproliferative neoplasms: life expectancy and implications of JAK2V617F in the occurrence of complications. Blood Cells Mol Dis. 2012;49(3-4):170-6.
39. Prasad S, Bala S, Chennamaneni R, Lakshmi KM, Sadashivudu G. Chronic myeloid leukemia in mother and son: What are the odds? Pediatr Blood Cancer. 2019;66(8):e27772.
40. Tsumura AM, Druker BJ, Brewer D, Press R, Braun TP. BCR-ABL+ Chronic Myeloid Leukemia Arising in a Family With Inherited ANKRD26-Related Thrombocytopenia. JCO Precision Oncology. 2021(5):415-7.
41. Verrou E, Tsirou K, Karampatzakis N, Triantafyllou T, Sevastoudi A, Grigoriadou GI, et al. Chronic myelogenous leukemia occurring in two brothers: The opposite sides of the same coin? Leuk Res Rep. 2021;16:100261.
42. Aitken MJL, Benton CB, Issa GC, Sasaki K, Yilmaz M, Short NJ. Two Cases of Possible Familial Chronic Myeloid Leukemia in a Family with Extensive History of Cancer. Acta Haematol. 2021;144(5):585-90.
43. Bondare DK, Teĭlane I, Reĭskart LV, Rotsena A, Grasmane DV. [Familial myeloproliferative syndrome (study of 4 families and review of the literature)]. Ter Arkh. 1985;57(7):59-64.
44. Kuan JW, Su AT, Wahab M, Hamdan A, Hashim J, Kiyu A, et al. The epidemiology of haematological cancers in Sarawak, Malaysia (1996 to 2015). BMC Cancer. 2023;23(1):563.
